# Synthesis of Polyaniline/Scarlet 3R as a Conductive Polymer

**DOI:** 10.3390/polym12030579

**Published:** 2020-03-05

**Authors:** Takuya Yonehara, Hiromasa Goto

**Affiliations:** Department of Materials Science, Faculty of Pure and Applied Sciences, University of Tsukuba, Tsukuba, Ibaraki 305-8573, Japan; s1820417@s.tsukuba.ac.jp

**Keywords:** polyaniline, scarlet 3R, CIE, doping

## Abstract

Polyaniline (PANI) was prepared in the presence of the acidic dye scarlet 3R. Color tuning was performed on PANI through doping–dedoping processes and by changing the solvent used during the optical absorption spectroscopic measurements. The chemical structure of the resulting polymer–dye composite was analyzed using infrared absorption spectroscopy, and it showed the occurrence of secondary doping in *m*-cresol. The shape of the UV–Vis optical absorption spectra for the composite solution is dependent on the types of organic solvents used during the analysis, which was influenced by the conformation of PANI and the ionic interactions between PANI and scarlet 3R.

## 1. Introduction

Polyaniline (PANI) is a promising conductive polymer whose unit molecule, aniline, is popularly used as a raw material for dyestuff, pigments, and medicine [1,2]. Composite materials using PANI are based on the conductivity of polymers; therefore, those combined with graphene [3] and magnetic materials [4] have been reported. Physical properties of giant magnetoresistance [5] and negative permittivity [6] for PANI have been studied. PANI that is synthesized via aniline polymerization is stable in air and exhibits moderate electrical conductivity, which is derived from both ionic and electrical conduction in the π-conjugation of the main chain. Its synthesis is basic and convenient when compared with the synthetic route undertaken by other conductive polymers in the sense that there is no need to use inert gas or organic solvent during the preparation process. Synthesis of PANI composites with inorganic materials such as TiO_2_ via emulsion polymerization yields a product (PANI) with high thermal stability [7]. PANI synthesis is generally conducted in an aqueous medium with the addition of an oxidizer as a polymerization initiator under acidic conditions. The anticorrosion function of PANI for metals has been further developed for applications [8].

Conductive polymer color tuning is often performed by adding dye; however, this process is difficult to perform because conductive polymers inherently possess a comparatively deeper, richer color than most dyestuff on the market today owing to their extensive π-electron system. Despite this disadvantage, conductive polymer–dye composites are still extremely valuable for practical applications in daily life. In this research, the synthesis of PANI, in the presence of an acidic dye, namely, scarlet 3R, was performed via doping (oxidation) and dedoping (reduction) processes. Changes in the electronic state and color of PANI upon application of the dye are discussed as the acidic dye used in this study partly functions as a surfactant, and reduction using ammonia/water further serves to remove the dye from the polymer.

## 2. Experimental Section

### 2.1. Synthesis

#### PANI-3R(ES)

**Scheme 1:**
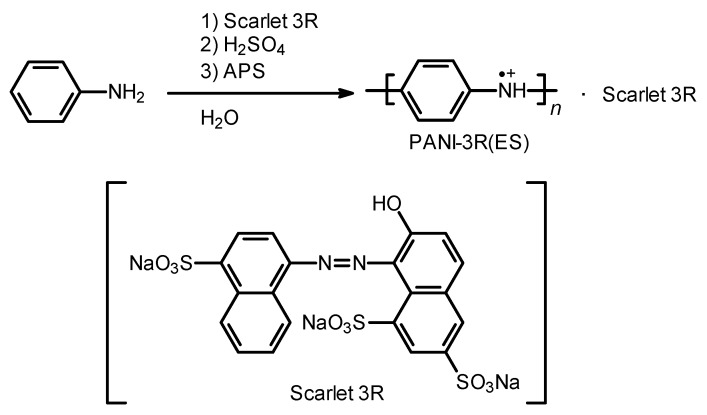
Synthesis of polyaniline-scarlet 3R-emeraldine salt (doped form) (PANI-3R(ES)). APS = ammonium persulfate.

**Figure 1:**
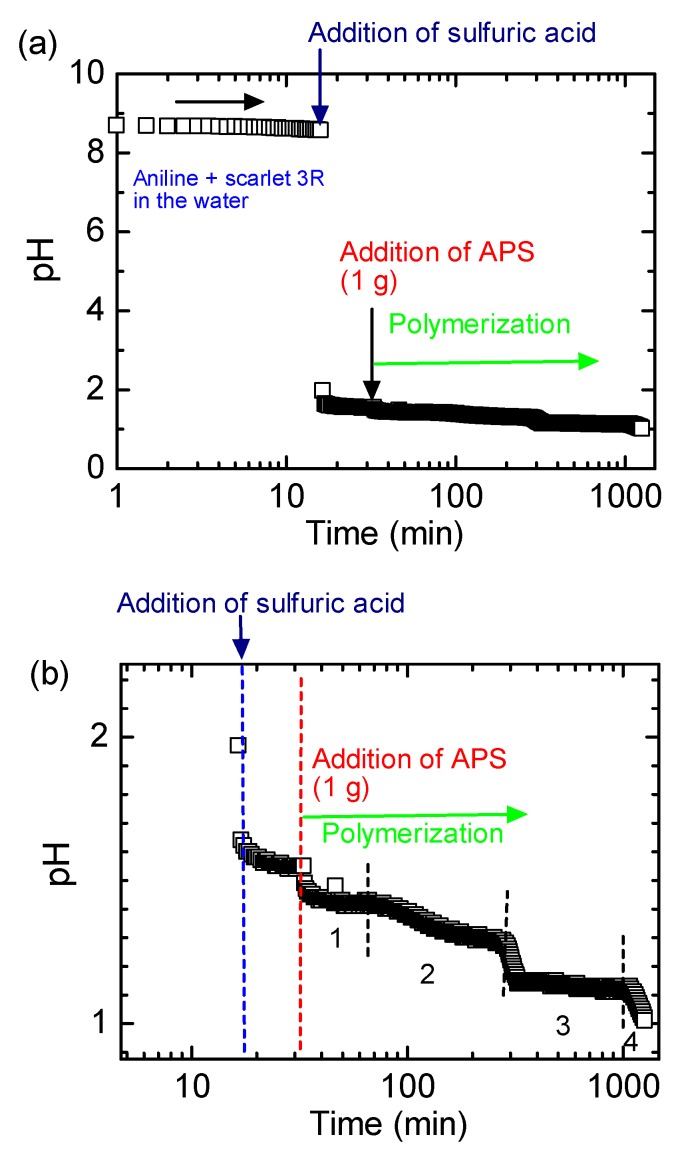
Polymerization profile. (**a**) Change in pH as a function of polymerization time. (**b**) Magnification of (**a**) after addition of sulfuric acid.

The preparation of polyaniline in the presence of scarlet 3R was performed with the aid of ammonium persulfate (APS) as an oxidizer (Scheme 1). First, scarlet 3R (2 g) was added to 100 mL of water at ca. 0 °C, followed by the dissolution of 2 g of aniline. Sulfuric acid (2 g) was then added, and this resulted in a rapid decrease in the pH of the solution as shown in Figure 1a. APS was subsequently added. As shown in Figure 1b (magnification of Figure 1a), 4-step pH changes of the reaction mixture were observed. After approximately 20 h, the reaction mixture was filtered, the resultant polymer residue was dried under reduced pressure, and it yielded 1.32 g of the desired product. The resulting polymer–dye in its prepared form is abbreviated as PANI-3R(ES), where ES denotes emeraldine salt. In this polymerization, scarlet 3R needs to be added to aniline in the water to form an aniline/dye complex prior to the addition of sulfuric acid in the synthesis. Preparation of PANI with the normal method was performed for comparison. The quantity of the chemicals for the synthesis is the same except for use of no scarlet 3R (Y = 0.802 g). The PANI prepared with the normal method is abbreviated as PANI_norm_.

### 2.2. Reagents and Methods

#### 2.2.1. PANI-3R(EB)

**Scheme 2:**
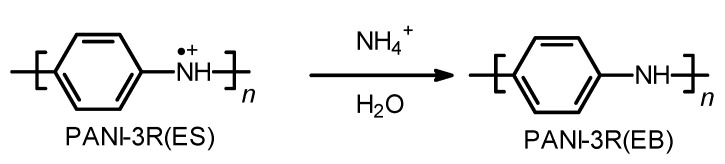
Synthesis of polyaniline-scarlet 3R-emeraldine base (reduced form).

An emeraldine base form of PANI-3R was prepared by treatment with an ammonia/water solution. PANI-3R(ES) (10 mg) was dissolved in 0.1 M of the ammonia/water solution and was stirred for 1 h. The resulting polymer slurry was filtered, and the residue was dried under reduced pressure to give the reduced form of the polymer, which is abbreviated as PANI-3R(EB) (Scheme 2). Here, EB denotes emeraldine base.

#### 2.2.2. Chemicals

Scarlet 3R was purchased from Takiguchi Shoten Co. (Tokyo, Japan). Aniline and APS were obtained from YONEYAMA KAGAKU KOGYO KAISHA, LTD. (Osaka, Japan) and used as received.

#### 2.2.3. Infrared (IR) Absorption Measurement

Each of the samples was measured using the KBr pellet method. The powdered sample (the amount is just enough to cover the tip of a spatula) was mixed with KBr. The pellet was then prepared using a hand press. The mixture of the sample powder and KBr was pressed to form a thin and transparent pellet.

#### 2.2.4. Thermogravimetric (TG) Analysis

TG analysis was performed on PANI samples. The samples were set in a platinum pan and heated to 600 °C at a rate of 10 °C/min under an argon atmosphere with an Ar–gas flow rate of 200 mL/min.

#### 2.2.5. X-ray Diffraction (XRD) Spectroscopy

The powdered sample of PANI was analyzed at room temperature. CuKα (*l* = 1.5428 Å). The XRD signals were directly interpreted from the 2*θ* value.

#### 2.2.6. Instrumentation

Infrared (IR) absorption spectra were obtained using an FT/IR-4600 spectrometer (Jasco, Tokyo, Japan) by the KBr method. UV–Vis absorption spectra were measured using a V-630 UV–Vis optical absorption spectrometer (Jasco, Tokyo, Japan). Electron spin resonance (ESR) measurement of the solid sample packed into a 5-mm quartz tube was performed using a JEOL JES TE-200 spectrometer in X-band (9.2–9.9 GHz) (JEOL, Tokyo, Japan). The measurement of electrical conductivity was performed using a Lowrester-GP and MCP-TP06P probe by the four-probe method (Mitsubishi, Tokyo, Japan). Scanning electron microscopy (SEM) observations were performed with JSM-7000F (JEOL, Akishima, Japan). Thermogravimetric analysis (TGA) was performed with an EXSTAR7000 (Seiko Instruments Inc., Chiba, Japan). IR absorption spectra were obtained with a JASCO FT-IR 550 spectrometer (Hachioji, Japan). XRD of the samples was measured with PANalytical X’Pert X-ray diffractometers (Almelo, The Netherlands).

## 3. Results and Discussion

### 3.1. FTIR

**Figure 2:**
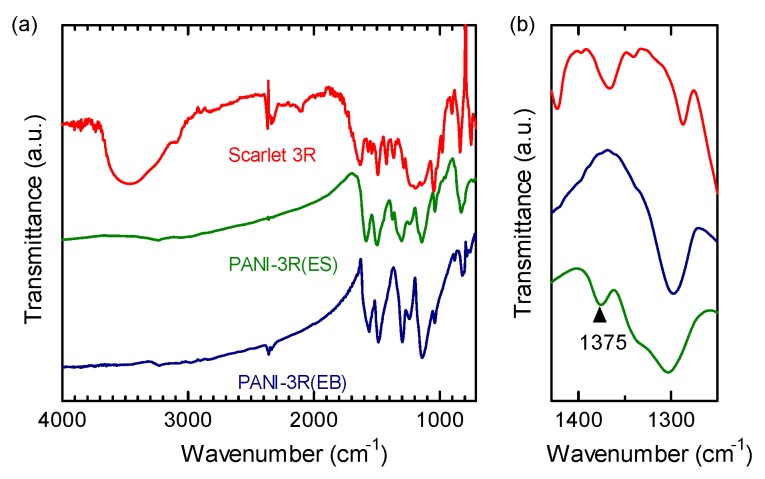
Infrared absorption of polyaniline-scarlet 3R-emeraldine salt (doped form, PANI-3R(ES)), polyaniline-scarlet 3R-emeraldine base (half-doped form, PANI-3R(EB)), and scarlet 3R (**a**). Magnification of the spectra (**b**).

**Figure 3:**
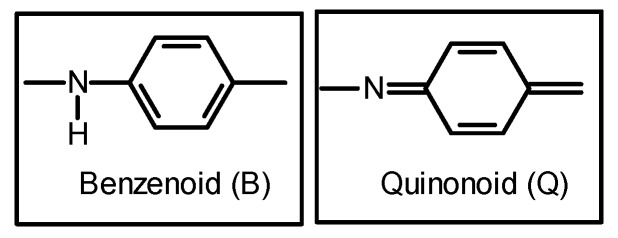
Chemical structures of Benzenoid (B) and Quinonoid (Q) in polyaniline.

Fourier-transform infrared (FTIR) spectroscopy was conducted with the KBr method as part of the chemical and structural characterization procedure (Figure 2a). As part of the reduction process, treatment of the polymer with ammonia yielded the half-doped state in which PANI-3R(EB) was partly doped with the residual ion. The result of the analysis revealed that PANI comprised a sequence of quinonoid (Q) and benzenoid (B) structures along the main chain (Figure 3).

The assignment of the functional groups and elements in the resultants was performed following the reported literature [9]. An absorption band due to NH_2_ stretching was observed at 3246 cm^−1^. Both PANI-3R(ES) and PANI-3R(EB) displayed N=Q=N and N–B–N stretching vibrations. PANI-3R(EB) had no absorption bands due to B–Q–B stretching or C–N stretching in BQB, QBB, and BBQ. It was proposed that the absorption band at around 1140 cm^−1^ is due to B–N^+^H=Q and B–N^+^H–B in PANI-3R(EB), which indicates that the partial doping of the polymer was due to absorption. Here, amino cations (N^+^) appeared in the doped state due to the removal of one electron from the lone pair of electrons on PANI’s nitrogen atom. Other absorption bands observed in the composite product are summarized in Table 1. An absorption band at 1041 cm^−1^ for polymers and scarlet 3R is observable. PANI-3Rs and PANI prepared by the normal method show almost the same absorptions in the IR. The present IR analysis was unable to detect absorptions due to the scarlet 3R fraction in the component of the polymer. However, a weak absorption band at 1375 cm^−1^ was observed as a result of SO_3_^-^ absorption of the scarlet 3R fraction in PANI-3R(ES), while PANI-3R(EB) shows no absorption derived from scarlet 3R, as shown in Figure 2b (magnification).

**Figure 4:**
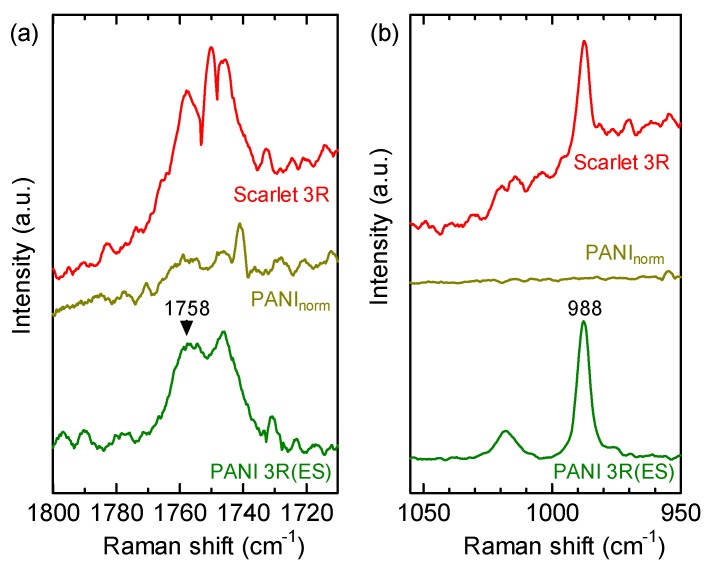
Raman scattering spectroscopy results for scarlet 3R, PANI_norm_, and PANI-3R(ES). (**a**) High-frequency region. (**b**) Low-frequency region (fingerprint region).

Figure 4 shows Raman scattering spectroscopy results for scarlet 3R, PANI_norm_, and PANI-3R(ES). PANI-3R(ES) showed Raman shifts at 1758 cm^−1^ (Figure 4a) and 988 cm^−1^ (Figure 4b) due to the signals from scarlet 3R, while PANI_norm_ did not show Raman shift signals. This observation revealed that PANI-3R contains scarlet 3R in the component.

### 3.2. Electron spin Resonance

**Figure 5:**
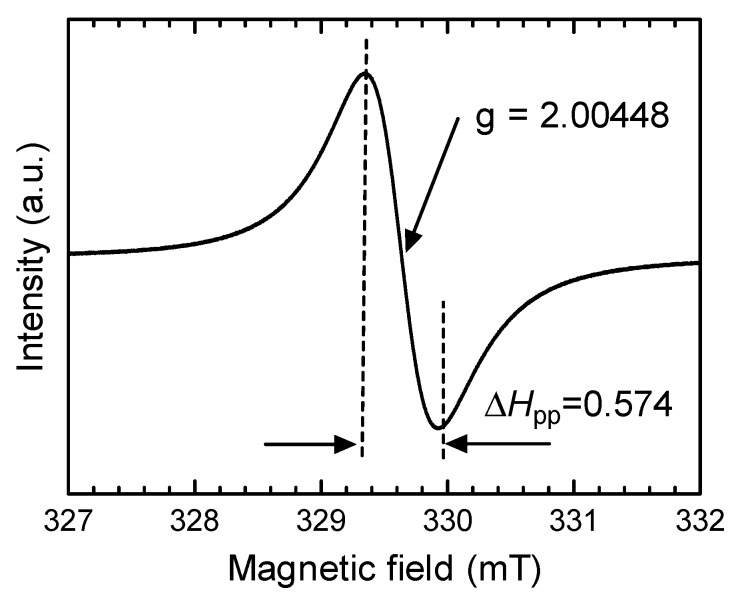
Electron spin resonance for PANI-3R(ES).

ESR analysis in the X-band was conducted for PANI-3R(ES) to confirm the presence of conduction electrons (commonly referred to as polarons). The ESR for PANI-3R(ES) was an asymmetric, Lorentz-type spectrum, which shows the presence of polarons (radical cations) that were delocalized along the main chain; this observation further confirms doping (Figure 5). The Δ*H*_pp_ (peak-to-peak line width) value for the polymer composite was relatively narrow (0.574 mT), suggesting the occurrence of charge carrier delocalization along the main chain. The g-value of the polymer composite proves that the charge carriers in this system were, indeed, polarons delocalized along polyaniline’s nitrogen–carbon sequence. The electrical conductivity of the pressed pellet form of PANI-3R(ES) as measured by the four-point probe method was 7.0 × 10^−1^ S/cm [5].

### 3.3. UV–Vis

**Figure 6:**
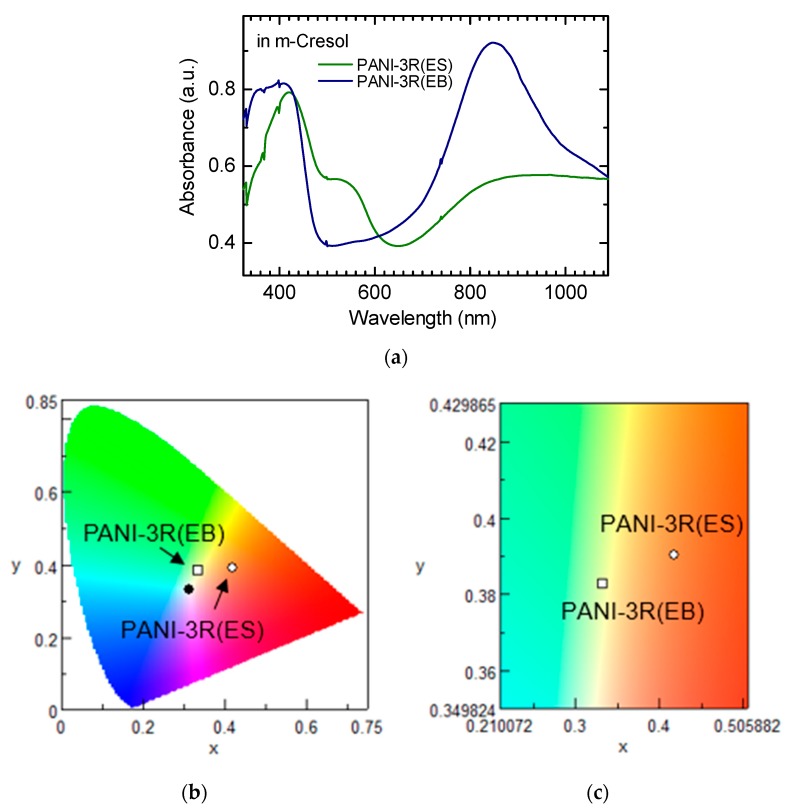
UV–Vis optical absorption spectra for PANI-3R(ES) and PANI-3R(EB) in *m*-cresol (**a**), the Commission Internationale de l’Éclairage (CIE) color spectrum (**b**), and magnification (**c**).

The polymers are soluble in *N*-methyl pyrrolidone (NMP), tetrahydrofuran (THF), and *m*-cresol. Ultraviolet–visible (UV–Vis) optical absorption spectroscopy was also performed on PANI-3R(ES) and PANI-3R(EB) in *m*-cresol solution, even though scarlet 3R possessed poor solubility in *m*-cresol. As seen in Figure 6a, PANI-3R(ES) and PANI-3R(EB) have absorption bands at long wavelengths due to the occurrence of secondary doping. The molecular conformation of PANI was changed from a compact to expanded coil as a result of secondary doping. MacDiarmid and Epstein et al. reported that secondary doping in the polymer allowed for the expansion of the effective π-conjugation length, particularly the extension of the absorption band for PANI-3R(ES) toward the red-infrared range [10,11]. The International Commission on Illumination (Commission Internationale de l’Éclairage, CIE) color spectrum identified PANI-3R(ES) as red in color and PANI-3R(EB) as yellow (Figure 6b,c).

**Figure 7:**
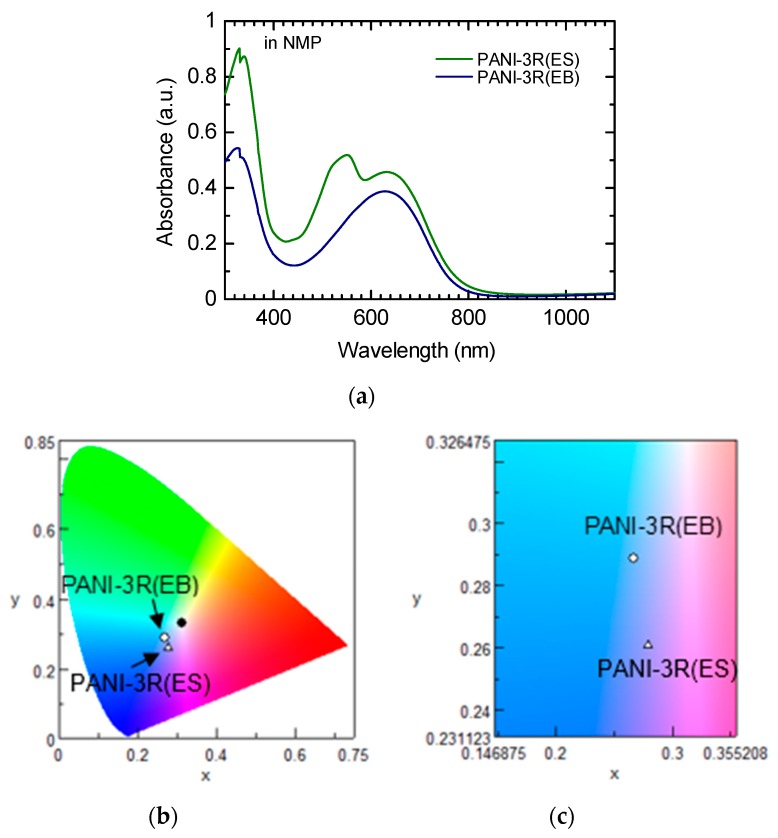
UV–Vis optical absorption spectra for PANI-3R(ES) and PANI-3R(EB) in *N*-methyl pyrrolidone (NMP) (**a**), the CIE color spectrum (**b**), and magnification (**c**).

**Figure 8:**
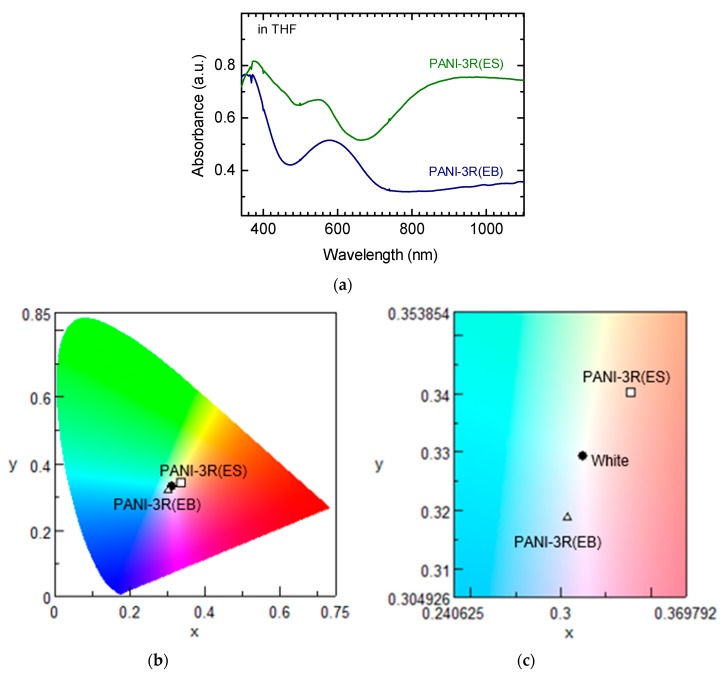
UV–Vis spectra for PANI-3R(ES) and PANI-3R(EB) in tetrahydrofuran (THF) (**a**), the CIE color spectrum (**b**), and magnification (**c**).

Figure 7a presents the UV–Vis spectra for PANI-3R(ES) and PANI-3R(EB) in NMP. The absorption bands at 521 nm (shoulder) and 547 nm were due to the absorption of scarlet 3R (Figure 8), indicating that PANI-3R(ES) formed a composite with the dye. Note that scarlet 3R is poorly soluble in *m*-cresol. On the other hand, NH_4_^+^ treatment of the PANI-3R(EB) composite resulted in the removal of scarlet 3R as shown by the lack of an intense absorption band related to 521 nm, although traces of absorption band were observed. No secondary doping occurred in NMP for any of the polymers. As the international standard model, the CIE color spectrum identified PANI-3R(ES) as having a purple-blue color and PANI-3R(EB) as being blue (Figure 7b,c).

Figure 8a presents the UV–Vis spectra for PANI-3R(ES) and PANI-3R(EB) in tetrahydrofuran (THF). The method used in our study allows for the synthesis of a THF-soluble PANI derivative, although the prepared PANI (i.e., in its doped form) is generally insoluble in organic solvents. PANI-3R(ES) displayed strong absorption bands at long wavelengths, whereas PANI-3R(EB) showed a weak absorption band at longer wavelengths. An absorption band at 585 nm indicates the presence of the PANI–emeraldine base since PANI in THF does not experience secondary doping effects. An absorption band for scarlet 3R was not observed. The optical absorption due to scarlet 3R overlapped with the intense absorption of the polymer composites, which was derived from the π-conjugation along the main chain. The CIE color spectrum classified PANI-3R(ES) as red and PANI-3R(EB) as blue (Figure 8b,c). These results showed that the electronic state and various solvent effects served as a means of color tuning the polymer composite. Doping samples (as prepared) are located in the red region of the color scale.

**Figure 9:**
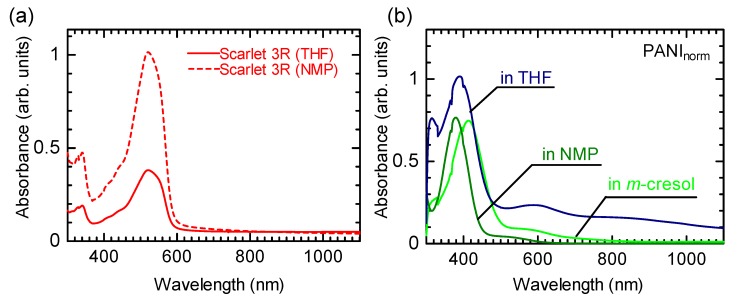
UV–Vis spectra for scarlet 3R in THF solution (red solid line), and scarlet 3R in NMP solution (red dashed line) (**a**), PANI_norm_ prepared with the normal method in THF, NMP and *m*-cresol (**b**).

Figure 9a shows UV–Vis spectra for scarlet 3R in THF and NMP solutions. Generally, the doped form of PANI (as prepared) has low solubility in organic solvents. Scarlet 3R is poorly soluble in *m*-cresol. Figure 9b shows soluble fractions of PANI_norm_ prepared with the normal method in THF, NMP, and *m*-cresol solutions. Absorptions at short wavelength of PANI_norm_ are a result of the π−π* transition of the benzene ring in the monomer repeat unit. Absorptions of PANI_norm_ at around 600 nm are due to the doping band. PANI_norm_ showed no absorption band at approximately 550 nm due to scarlet 3R, which confirmed that PANI-3Rs (PANIs prepared in the presence of scarlet 3R) with this absorption contain a scarlet 3R fraction in the component.

### 3.4. Scanning Electron Microscopy

**Figure 10:**
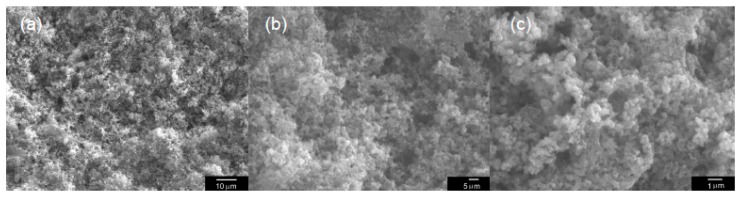
Scanning electron microscopy (SEM) images of PANI_norm_. (**a**) 1000×, (**b**) 5000×, (**c**) 7000×.

**Figure 11:**
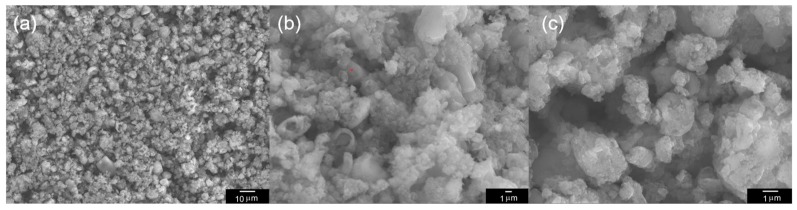
SEM images of PANI-3R(ES). (**a**) 1000×, (**b**) 3000×, (**c**) 7000×.

Figure 10a–c shows scanning electron microscopy (SEM) images of PANI_norm_ prepared with the general method. PANI_norm_ exhibits a short fiber structure due to molecular aggregation. Figure 11a–c shows SEM images of PANI-3R(ES). The polymer shows no fiber-like structure under the SEM. Globular and partly broken egg structures are observed (Figure 11b). The globular structures are formed during the polymerization process by the interaction between the monomer and the scarlet 3R.

### 3.5. XRD

**Figure 12:**
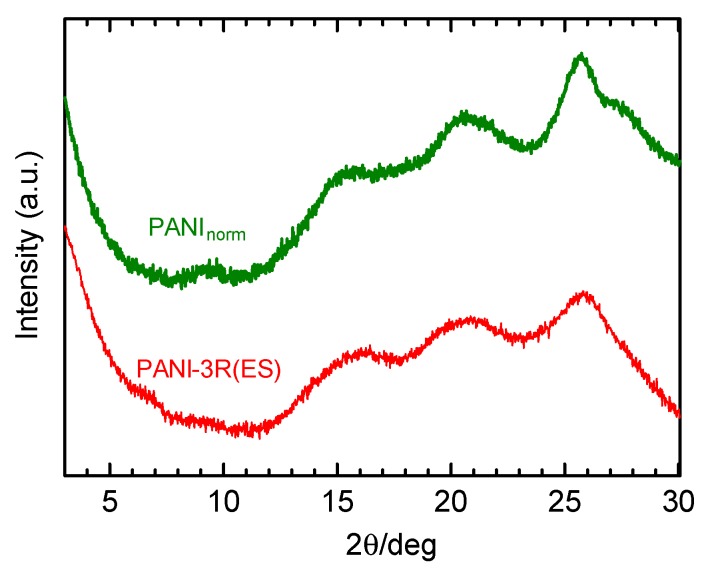
X-ray diffraction patterns of PANI_norm_ and PANI-3R(ES).

XRD analysis was performed for PANI_norm_ and PANI-3R(ES), as shown in Figure 12. The diffraction patterns of PANI_norm_ and PANI-3R(ES) agree with previous results [12]. PANI_norm_ shows four peaks at 2θ = 9.4, 15.6, 20.6, and 25.7°, while PANI-3R(ES) shows three peaks at 2θ = 15.9, 20.7, and 25.7° due to a decrease in crystallinity. The signal at 9.4° may be due to the inter-main chain distance. It was reported that PANI prepared in the presence of sodium dodecylbenzene sulfonic acid (SDBSA) as a surfactant shows no signal at 9.4° due to a decrease in crystallinity [13]. Similarly, in this study, a decrease in crystallinity compared to the PANI prepared with no surfactant was observed, which was attributed to the presence of scarlet 3R that acted as a surfactant for the synthesis.

### 3.6. Thermogravimetric Analysis

**Figure 13:**
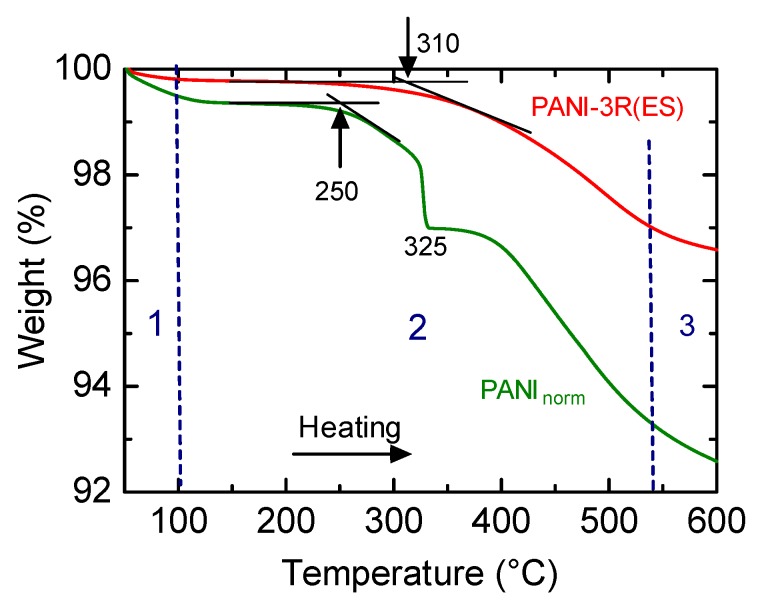
Thermogravimetric (TGA) curve of PANI-3R(ES). Scan rate: 10 °C/min. Region 1: water loss. Region 2: loss of dopant and the dye. Region 3: carbonization.

The results of the TGA of PANI-3R(ES) and PANI_norm_ are shown in Figure 13. In Region 1, there was an out-gassing of the sample in which the moisture was evaporated below 100 °C. The degradation begins at 250 °C (onset), resulting in the weight loss of PANI_norm_. Meanwhile, the thermal degradation of PANI-3R(ES) begins at 310 °C (onset), indicating PANI-3R(ES) has a higher thermal stability than that of PANI_norm_. The thermal decomposition of PANI-3R(ES) is gradual, and the magnitude of the curve increases with temperature. Loss of the dopants (hydrogen sulfate or the dye) and degradation of the polymer occur in Region 2 (Figure 13). PANI_norm_ shows drastic weight loss at 325 °C, which may be due to a loss of dopant (hydrogen sulfate). In comparison, PANI-3R(ES) exhibits no such drastic loss in the heating process. This may be due to the fact that the PANI being wrapped by the dye increases the thermal stability. Carbonization occurred in Region 3, as shown in Figure 13. The total loss weight of PANI_norm_ is 7.5% when heated to 600 °C, compared to 3.42% for PANI-3R(ES). This result confirms that the polymerization of aniline in the presence of the dye allows for the creation of PANI-dye material with an improvement in thermal stability.

### 3.7. Proposed Structure

**Figure 14:**
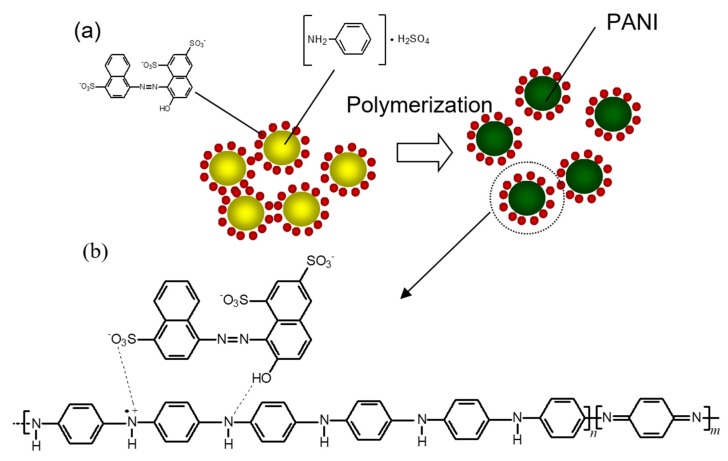
Formation of nanoparticles in the polymerization (**a**) and proposed structure for PANI-3R(ES) as a polymer–dye composite (**b**).

Pandiselvi et al. synthesized a chitosan–PANI/ZnO hybrid for the removal of orange 16 dye and indicated that PANI interacts with dyes [14]. N-ions in the PANI-3R(ES) (doped state, emeraldine salt form) with a positive charge (N^+^) interacted with the negatively charged scarlet 3R ions during the polymerization process (Figure 14a). Since it is an acidic dye, scarlet 3R functions as an oxidizer; the anionic portion of scarlet 3R bonded with PANI molecules via ionic interactions and functioned as a surfactant during the production of aniline monomer nanoparticles before the addition of APS for polymerization processes [15]. Therefore, the dye created a layer of nanospheres around the PANI that were similar to those seen when SDBSA acts as an anionic soap during PANI synthesis (Figure 14b). shows the interaction of PANI and scarlet 3R via ionic and hydrogen bonds. In addition, PANI partly interacts with sulfuric acid.

## 4. Conclusions

Colorization of conductive polymers is a very challenging task due to their intense inherent color caused by extensive π-conjugation along the main chain of these polymers. Despite this issue, the colorization of conductive plastics still has important real-world applications. In this study, PANI was prepared in the presence of the acidic dye scarlet 3R, which functions as a surfactant. By countering the ionic nature of PANI, scarlet 3R was able to form a PANI–dye composite via doping–dedoping (oxidation–reduction) processes and effective solvent selection. Herein, the first attempt at tuning the color of a conductive polymer with dyestuff was reported.

## Figures and Tables

**Table 1:** Assignment of IR spectra for PANI–dye composites [9].

PANI-3R(ES) (cm^−1^)	PANI-3R(EB) (cm^−1^)	Assignment
3246	3246	NH_2_ Str.
1588	1561	Str. of N=Q=N
1503	1489	Str. of N–B–N
1498	1484	Str. of benzene ring
1374	-	Str. of B–Q–B
1336	-	C-N str. in BQB, QBB, BBQ
1144	1144	B-N^+^H=Q, B–N^+^H–B
1039	1041	C–H in plane on 1,2,4-ring

Str. = stretching, Q = quinonoid, B = benzenoid.
